# Establishment and psychometric characteristics of emotional words list for suicidal risk assessment in speech emotion recognition

**DOI:** 10.3389/fpsyt.2022.1022036

**Published:** 2022-11-11

**Authors:** Juan Shen, Shuo Zhang, Yongsheng Tong, Xiangmin Dong, Xuelian Wang, Guanghui Fu, Liting Zhao, Mengjie Wu, Yi Yin, Yuehua Wang, Nancy H. Liu, Jianlan Wu, Jianqiang Li

**Affiliations:** ^1^Beijing Suicide Research and Prevention Center, Beijing Huilongguan Hospital, Beijing, China; ^2^WHO Collaborating Center for Research and Training in Suicide Prevention, Beijing, China; ^3^Faculty of Information Technology, Beijing University of Technology, Beijing, China; ^4^Peking University Huilongguan Clinical Medical School, Beijing, China; ^5^Sorbonne Université, Institut du Cerveau—Paris Brain Institute—ICM, CNRS, Inria, Inserm, AP-HP, Hôpital de la Pitié-Salpêtrière, Paris, France; ^6^Department of Psychology, University of California, Berkeley, Berkeley, CA, United States

**Keywords:** suicidal risk, emotional word, speech emotion recognition, hotline, reliability, validity

## Abstract

**Background:**

Emotional disturbance is an important risk factor of suicidal behaviors. To ensure speech emotion recognition (SER), a novel technique to evaluate emotional characteristics of speech, precision in labeling emotional words is a prerequisite. Currently, a list of suicide-related emotional word is absent. The aims of this study were to establish an Emotional Words List for Suicidal Risk Assessment (EWLSRA) and test the reliability and validity of the list in a suicide-related SER task.

**Methods:**

Suicide-related emotion words were nominated and discussed by 10 suicide prevention professionals. Sixty-five tape-recordings of calls to a large psychological support hotline in China were selected to test psychometric characteristics of the EWLSRA.

**Results:**

The results shows that the EWLSRA consists of 11 emotion words which were highly associated with suicide risk scores and suicide attempts. Results of exploratory factor analysis support one-factor model of this list. The Fleiss’ Kappa value of 0.42 indicated good inter-rater reliability of the list. In terms of criteria validities, indices of despair (Spearman ρ = 0.54, *P* < 0.001), sadness (ρ = 0.37, *P* = 0.006), helplessness (ρ = 0.45, *P* = 0.001), and numbness (ρ = 0.35, *P* = 0.009) were significantly associated with suicidal risk scores. The index of the emotional word of numbness in callers with suicide attempt during the 12-month follow-up was significantly higher than that in callers without suicide attempt during the follow-up (*P* = 0.049).

**Conclusion:**

This study demonstrated that the EWLSRA has adequate psychometric performance in identifying suicide-related emotional words of recording of hotline callers to a national wide suicide prevention line. This list can be useful for SER in future studies on suicide prevention.

## Introduction

Suicide is one of the leading causes of death worldwide, with 703,000 suicide deaths globally in 2019, and suicide accounts for 1.3% of all causes of deaths (1). Although in China, suicide rates have decreased year by year over the past few decades, from 18.1 per 100,000 in 1990 (2) to 6.7 per 100,000 in 2019 (1), still, approximately 100,000 persons in China died by suicide in 2019 (1).

Emotional disturbance, including hopelessness and depression, are important risk factors for suicidal behaviors (3). Negative emotions such as depression, anxiety, and despair, were more severe in students at risk for suicide than those students not at risk (4). Among people with depression, those reporting recent suicide attempts had greater psychological distress than those without (5). Anxiety disorders are also associated with an increased incidence of suicidal ideation and suicide attempts in adolescents and young adulthood (6).

To overcome potential concealments of real emotional status in rating depression and other emotions using common scales, novel techniques on facial expression and text content are commonly used. Severity of depression was correlated with facial expression features which were identified via movements of expression muscles (7). A machine learning model for the assessment of depression severity was built by extracting facial expression features (8). In study using lexical analysis of posts on social media, suicidal behavior was associated with high frequency of words of distress, fear, loneliness, despair, and sadness (9). Among adolescents, the words of psychological distress and despair were frequently used by those reporting depressive symptoms and suicidal behaviors (10). Results of quantitative analysis of semantic and emotional structure of suicide notes extracted key emotions of suicidal behaviors (11). However, text analyses might misclassify neutral discussions on suicide related issues as high risk, due to a lack of non-verbal behavioral information.

Speech Emotion Recognition (SER) establishes the association between speech features and emotions of the speaker (12). Individuals at risk for depression and suicide differ from normal controls in speech features such as speech rate, response time, fundamental frequency and the formants (13, 14). Many studies have argued that the SER model is accurate enough to classify different emotions (15, 16), and to identify changes in depression severity before and after remission in patients with depression (17). Indeed, many databases have been developed for emotion recognition, such as the IEMOCAP (18), the VAM (19), and the RAVDESS (20).

As yet, existing SER databases have not labeled suicide-related emotion categories (21, 22). As a result, these databases may not be useful for establishing a prediction model for subsequent suicidal behaviors. Therefore, what needed is a modified SER model specific to suicidal behavior prediction established via a prospective study. Furthermore, previous studies on facial expression and text analyses (7–10) utilize cross-sectional designs, which limits the ability to predict subsequent suicidal act.

In order to establish a suicidal behavior specific SER model, the first step in this study therefore, is to develop a suicide-related emotional words list for labeling speech characteristics of hotline calls (23). This kind of emotional word list could be used in future artificial intelligence (AI)-based SER model for suicidal risk assessment.

The second step would be validation of the list. Psychological hotline is an effective measure for suicide prevention (24, 25) and provides the ideal material for suicide-related speech. In this study, we validate the Emotional Words List for Suicidal Risk Assessment (EWLSRA), through establishing its psychometric properties, namely inter-rater reliability, construct validity, and criterion validity, using speech materials collected at a big hotline in China. In this step, association of speaker’s emotional status labeled by the EWLSRA and subsequent suicidal acts would be tested using a prospective design.

## Methods

### Establishment of the emotional words list for suicidal risk assessment

Based on their professional knowledge in suicide prevention, 10 professionals independently identified an unlimited number of emotional words related to suicidal behavior. After this stage, the 10 professionals discussed together and reached consensus about the remaining emotional words and putting them into several categories. At the end of this, a final list of suicide-related emotional words was established.

### Reliabilities and validities of the emotional words list for suicidal risk assessment

#### Materials and instruments

**Tape-recordings** of 68 calls to the Beijing Psychological Support Hotline was selected by convenience sampling. Among them, three were for training how to label the emotional status of the caller, 2 recordings (consisted of 129 valid segments) were used for testing inter-rater reliability, and the remaining 63 recordings were used for establishing criterion validity. The inclusion criteria of the recordings included the following: (a) callers seeking for help for their own psychological problems; (b) callers for whom data on demographic variables and psychosocial characteristics were completed. Excluded recordings were those that were invalid calls, e.g., silence, harassment, or calls of less than 10 min (most of these calls missed data on suicidal risk score and subsequent suicidal acts, details were described below).

**Adobe Audition CC 2018** was used to cut all recordings into segments. Each segment comprises only one speaker’s speech, i.e., any conversation between two or more speakers were further cut apart.

**Mate Mark** is a software developed by our team. The software was used to label emotional words on each segment of included tape-recordings. While a tape-recording was loaded into the Mate Mark, each segment, accompanied by several buttons, was presented on the screen. The buttons consisted of speaker options (caller or others), 11 emotional words in the EWLSRA as established in this study, one “null” button (indicating those without any suicide-related emotion), and buttons of “invalid” and “unclear.” A trained researcher labeled at least one of the 11 emotional words, except for three conditions: (1) segment was labeled “null,” (2) the researcher selected “others” button in the speaker options (indicating the segment was speech of someone other than caller to hotline), and (3) the segment was labeled “invalid” or “unclear,” which indicated that the segment was too noisy or otherwise indecipherable. Those segments of speech (other than those labeled as other callers, invalid, or unclear) without any labels of emotional words were excluded in final data analysis.

#### Questionnaires

**The EWLSRA** was slightly modified as a questionnaire. In the questionnaire, 11 emotional words in EWLSRA were listed, and participants (described below) were required to evaluate the level of association between each emotion described by the word and suicidal behavior. A 7-point Likert scale was used, with 1 indicating no association and 7 indicating the greatest association.

**The Suicidal Risk Assessment Scale** has been developed to predict subsequent suicidal acts among hotline callers and was used in a previous study (26). The scale consists of 12 items, including suicidal ideation and plan, severity of depression, etc. Total score of the scale ranges from 0 to 16, with higher scores indicating higher risk for subsequent suicidal acts. In this study, the suicidal risk score was used to examine the criterion validity of the EWLSRA.

#### Subsequent suicidal acts in 12 months

In a previous study, many callers with low-, moderate, or high-risk for subsequent suicidal acts were scheduled for follow-up (26). The purpose of the follow-up is to investigate who would make suicidal acts (attempt or death) after the intake call. In each scheduled follow-up, research assistants would ask callers “Did you attempt suicide after (the date of index call or the last follow-up)?” If caller answered “yes,” assistant would ask details of the act to clarify whether it was suicide attempt. If the caller cannot be reached, the family member answering the phone would be inquired whether the caller has had fatal suicidal behavior. In this study, a follow-up time period of 12-month was set, because most of the suicidal acts occurred during the first 12-month (27). Occurrence or absence of suicidal acts in the 12-month after index call were recorded in the database. In this study, we collected data on subsequent suicidal acts that occurred in the 12-month after index call in order to test the predictive validity of the EWLSRA.

#### Participants

Seven researchers with experience in suicide prevention were trained on how to label emotional words for each segment of recording. To test inter-rater reliability of the EWLSRA, they were required to independently label no more than one emotional word for each segment of two calls’ tape-recordings. To examine the construct validity of the EWLSRA, we recruited doctors, nurses, psychotherapists, and postgraduate students in psychiatry or psychology from August to September in 2021 to complete a questionnaire. In total, 201 respondents were recruited and completed an online questionnaire.

### Procedure

This study was administered as follows: First, we established the EWLSRA, which was obtained via expert identification, nomination, and consensus discussion. Second, 7 researchers were trained in labeling the emotion words for each segment, which allowed us to explore the inter-rater reliability of the EWLSRA. Third, 7 trained researchers labeled emotional words on segments of 63 selected call recordings. For each recording, three researchers labeled it independently. Fourth, two main outcomes (total suicidal risk score and subsequent suicidal acts) of the 63 callers whose call recording were used to evaluate the criterion validity of the EWLSRA. All researchers were blind to the outcomes while they labeled suicide-related emotional characteristics of selected calls’ tape-recordings. Fifth, an online data collection using Wenjuanxing (an app for online data collection widely used in mainland China) was conducted to examine the construct validity of EWLSRA.

### Statistical analysis

Data analyses were performed using SPSS version 26.0. All segments labeled as speakers other than hotline callers, invalid, or unclear were excluded in final data analysis. Inter-rater reliability was estimated using 129 “valid” segments (details were described above) of 2 call recordings. Fleiss’ Kappa was used to estimate inter-rater reliability.

Criterion validity: Indices of 11 emotional words were calculated in this study. For each included tape-recording, the index of any suicide-related emotional word of the EWLSRA is the total numbers of the specific word labeled by 3 researchers, divided by numbers of remained “valid” segments of the recording, and multiply by 100. Two criteria were used to test criterion validity separately. Spearman’s rank correlation coefficients were calculated between the indices of the 11 emotional words and caller’s suicidal risk score. Secondly, based on the occurrence of subsequent suicidal acts in the follow-up period, the 63 callers were divided into two sub-groups: one with suicide or suicide attempt and the other one without. Wilcoxon rank sum test was used to compare difference of indices of 11 emotional words in the EWLSRA between the two sub-groups.

Exploratory factor analysis was run to examine construct validity of the EWLSRA.

### Ethics approval statement

The present study was approved by the Institutional Review Board of the Beijing Huilongguan Hospital (2021-16-science). The authors assert that all procedures contributing to this work were conducted in accordance with the ethical standards of the Helsinki Declaration as revised 1989. Because existed tape-recording dataset was used in this study, and any personal information has been deleted prior to data-analysis, written informant consents have been waived.

## Results

### Establishment of the emotional words list for suicidal risk assessment

The 10 professionals who nominated emotional words have engaged in suicide prevention for 2–17 years. Their demographic characteristics are listed in the Table 1. After nomination and discussion, the final list consisted of 11 emotional words: despair, sadness, psychological distress, guilty, confusion, helplessness, resentment, fear, numbness, anxiety, and grievance (see Table 2). Interpretations of each emotion word are shown in Supplementary material.

**TABLE 1:** Demographic characteristics of the 10 professionals.

	Gender	Title	Years of engaging in suicide prevention
1	Male	Professor	17
2	Female	Researcher	6
3	Female	Internship	6
4	Female	Internship	3
5	Female	Resident	3
6	Female	Resident	4
7	Female	Internship	2
8	Female	Resident	3
9	Female	Psychotherapist	14
10	Female	Internship	2

**TABLE 2:** Details of the EWLSRA and loadings of 11 suicide-related emotional words in the one-factor model among 201 respondents.

Emotion categories	Synonym or similar words	Factor loading
Despair (绝望)	Hopelessness (无望), entrapment (围困感)	0.47
Sadness (悲伤)	Depression (抑郁, 消沉, 沮丧, 沉闷, 忧伤, 低落), dejected/dispirited (消沉, 沮丧), pessimism (悲观), negative (消极), gloom (郁闷, 忧郁), sorrow (悲哀, 哀伤,伤心), grieve (哀伤), sense of loss (失落), distressed (忧愁, 苦恼, 忧虑, 烦闷), decadence (颓废), sentimental (伤感), upset (不悦, 难过)	0.72
Psychological Distress (痛苦)	Breakdown (崩溃), repression, oppressive (压抑), aggrieve (悲痛, 沉痛, 痛心), suffocation (窒息), stifle (压抑, 窒息)	0.71
Guilty (负罪感)	Shame (羞辱感, 惭愧), lose status (丢脸), self-reproach (自责, 自罪自责, 自怨自艾), guilt (罪恶感, 内疚), 愧疚), regret (遗憾, 后悔), self-hate (自厌), low self-esteem (自我贬低), inferiority (自卑)	0.69
Confusion (迷茫)	At a loss (不知所措, 迷惘, 彷徨, 茫然), overwhelming (无奈)	0.79
Helplessness (无助)	Loneliness/desolate (孤独, 孤寂, 落寞, 寂寞), isolation (孤立)	0.74
Resentment (愤恨)	Indignation (恼怒, 气愤), complain (抱怨, 埋怨), grudge/resentment (怀恨, 怨恨), disgust/hate (厌恶, 痛恨), rage/fury (暴怒, 狂怒), anger (生气, 愤怒)	0.72
Fear (恐惧)	Dread (畏惧, 害怕, 畏怯, 胆怯), panic (恐慌, 慌张), flustered (慌张), terrified (惶恐, 害怕), scare (害怕, 惊恐, 惊慌)	0.76
Numbness (麻木)	Meaninglessness (空洞, 虚妄, 空虚), indifferent (无所谓), bored (无聊)	0.63
Anxiety (焦虑)	Irritable/fidget (烦躁), nervous (紧张), restless (焦躁, 不安)	0.71
Grievance (委屈)	Treat unjustly (冤枉, 憋屈), dishonor (丢面子)	0.63

### Reliabilities and validities of the emotional words list for suicidal risk assessment

#### Construct validity

In the exploratory factor analysis, the KMO value was 0.90, and Bartlett’s spherical test χ*^2^* = 910.88, *P* < 0.001. One-factor model was identified, because only one factor demonstrated an eigenvalue > 1. The model explained 48.0% of the variance, and the loadings of 11 items were all > 0.4 in this model (see Table 2).

#### Inter-rater reliability

The inter-rater reliability of the EWLSRA was moderate agreement (28), with the Fleiss’ Kappa value of 0.42.

#### Criterion validity

Of the 63 Chinese callers whose tape-recorded calls were included in this study, 27 (43%) were males and 36 (57%) were females, the average age of the callers were 24.71 years old (SD = 7.41; range 12–43). Fifty-four callers (23 males and 31 females) obtained scores of the suicidal risk assessment scale. Correlation analysis showed that indices of 4 emotional words in the EWLSRA were statistically significantly associated with scores of suicidal risk assessment scales, they were despair (Spearman ρ = 0.54, *P* < 0.001), sadness (ρ = 0.37, *P* = 0.006), helplessness (ρ = 0.45, *P* = 0.001), and numbness (ρ = 0.35, *P* = 0.009) (see Table 3).

**TABLE 3:** Criterion validity analysis of the indices of 11 suicide-related emotional words in the EWLSRA.

Emotional words	Median (IQR)			Mann-Whitney rank sum test^a^		Spearman’s correlation^b^	
			
	Total (*N* = 63)	Without suicide attempt (*n* = 28)	With suicide attempt (n = 20)	*Z*	*P*	ρ	*P*
Despair	1.48 (0.00–3.93)	0.92 (0.00–3.30)	2.07 (0.51–4.09)	−1.65	0.10	0.54	<0.001
Sadness	23.00 (9.34–37.39)	16.85 (6.80–35.85)	22.81 (14.09–39.53)	−0.94	0.35	0.37	0.006
Psychological distress	4.85 (2.41–15.43)	4.62 (1.23–28.10)	5.86 (3.76–16.81)	−0.52	0.60	0.30	0.03
Guilty	1.31 (0.00–4.65)	1.29 (0.08–4.31)	1.05 (0.00–6.35)	−0.23	0.82	0.20	0.14
Confusion	5.19 (2.13–10.49)	5.14 (2.55–10.91)	4.77 (1.73–10.34)	−0.75	0.45	0.07	0.61
Helplessness	3.54 (1.12–8.66)	3.43 (1.22–12.01)	3.28 (0.57–5.45)	−0.71	0.48	0.45	0.001
Resentment	1.87 (0.00–6.43)	2.16 (0.00–9.24)	0.88 (0.08–4.34)	−0.03	0.98	0.04	0.77
Fear	0.00 (0.00–1.06)	0.00 (0.00–0.59)	0.00 (0.00–1.37)	−0.66	0.51	0.15	0.27
Numbness	1.94 (0.00–3.63)	1.06 (0.00–2.69)	2.81 (1.21–5.07)	−1.96	0.049	0.35	0.009
Anxiety	1.50 (0.47–6.27)	2.17 (0.64–8.49)	0.93 (0.00–2.45)	−1.85	0.07	−0.16	0.25
Grievance	2.61 (0.57–11.64)	2.31 (0.48–9.23)	3.23 (0.46–13.45)	−0.36	0.72	0.29	0.04

^a^Indices of 11 suicide-related emotional words and comparisons of the indices between callers with or without suicide attempt in 12 months, using Wilcoxon Mann-Whitney rank sum test.

^b^Spearman correlation coefficients of the indices of 11 suicide-related emotional words and scores of suicidal risk assessment scales among 54 tape-recorded calls.

Of the 63 included callers, 48 callers were followed at least once in the 12 months after the index call. Among them, 0 died by suicide, 20 reported suicide attempts, and 28 did not report any suicidal act. The difference of index of the emotional word numbness between the callers with and without suicide attempt was statistically significant (*P* = 0.049) (see Table 3).

## Discussion

### Main findings

In this study, we established and tested the psychometric characteristics of the EWLSRA. Results indicated that the final EWLSRA consisted of 11 emotional words and was able to distinguish the emotional speech characteristics of calls obtained from a large hotline for suicide prevention in China. The EWLSRA could be used to label speaker’s suicide-related emotional status, which is prerequisite in developing AI-based SER model for suicide prediction.

Our findings indicated that the callers with high risk for suicidal behaviors, e.g., with higher scores of suicidal risk assessment scale or with occurrence of suicide attempt in the 12-month follow-up, were more frequently labeled emotional words in the EWLSRA, including despair, sadness, psychological distress, helplessness, numbness, and grievance. This is similar to previous study using lexical analysis, in which microblog users who died by suicide pasted more negative emotional words than those without suicidal ideation (29). A large scale of previous studies have identified that individuals reporting suicidal ideation or suicide attempts were more likely to experience depressive symptoms (30), psychological distress (31), helplessness and loneliness (32, 33), and feelings of numbness or detachment from others (34). All of the forementioned negative emotions were described by the EWLSRA developed in our study. Results in this study emphasized that these suicide-related emotions were expressed in speech and sentence characteristics and could be recognized well.

Among the 11 emotional words in the EWLSRA, guilty, confusion, resentment, fear, and anxiety were not associated with suicidal risk scores or occurrence of subsequent suicide attempts. Many researchers have argued that some negative emotions, such as anxiety, are associated with suicidal behavior through the mediating effects of other negative emotions, for example, despair and depression (35). Considering the high factor loads of these emotional words in our study, we did not delete these five words from the list. To refine the EWLSRA, we will test the associations of these words and suicidal behaviors in future studies.

The inter-rater reliability of the 7 researchers was in moderate agreement (28), with a Fleiss’ Kappa value of 0.42. Considering 7 researchers participated in the test and no more than one of the 11 emotional words could be used to label one given segment, this moderate agreement appears acceptable and indicates that the EWLSRA is reliable for several professionals to independently label suicide-related emotional speech characteristics, a key process of SER.

Results of the exploratory factor analysis demonstrated a one-factor model of the EWLSRA. To some extent, this might reflect that all of the 11 emotion words are closely associated with one potential latent variable. Given the content of the 11 emotional words included in the EWLSRA, we argue that this latent variable as a suicide-related emotional characteristic.

Many previous studies have focused on lexical or content analysis for suicide notes or messages posted online by persons with suicidal ideation or suicide attempts, who died by suicide, or were at-risk for suicidal behaviors (10, 11, 36–38). In China, a Chinese suicide dictionary was developed for content analysis using social media materials (Sina Weibo, a popular microblog in mainland China). The suicide dictionary contains 2,168 words that can be classified into 13 different categories, such as suicidal ideation, suicide methods, hopelessness, and somatic complaints (39). However, this dictionary was not developed for the description of emotional speech characteristics of individuals reporting suicidal behaviors, and it may not suitable to be used for suicide-related emotional speech recognition.

Compared with the lexical and content analysis, the SER model includes additional information of speech features. Although most emotional words in the EWLSRA were also embodied in many existing rating scales for negative emotion (3, 5, 6), few of previous studies verified whether these words could be used to describe emotional speech characteristics. To the best of our knowledge, this study is the first to establish an emotional words list for suicidal behaviors, based on real-world speech material in a national suicide prevention hotline. The EWLSRA is useful for labeling suicide-related emotional speech characteristics, which might be a key component in future studies for developing effective AI-based evaluation methods for risk identification in suicide prevention.

### Limitations

There are some limitations in this study. First, some suicide-related emotional words may not have been included in this study. Second, the sample size was small, only 63 tape-recordings were included for evaluating criterion validity, and 129 segments of 2 recordings were included for inter-rater reliability test. If a larger sample with prospective follow-up data was recruited, it would improve the capacity for refining the reliability and validity of the EWLSRA. Third, the speech materials labeled in this study were obtained from one suicide prevention hotline, which might limit the generalization of our findings to other settings. Fourth, the emotional words included in the EWLSRA are Chinese, and only mandarin speaking calls were used to verify validities of the EWLSRA, it limits generalization of the list to countries with different cultural background. The psychometric characteristics of the EWLSRA should be re-verified while researchers in other countries try to carry out early identification of suicidal risk based on SER model. Fifth, as reported in previous study (40), predictive accuracies would be improved with shorter interval between index assessment and occurrence of main outcome, however, in our study we have to select an interval of 12-month to validate predictive value of the list, due to low base rate of subsequent suicide attempts. A long interval would limit upper bounds of predictive value of this list. Sixth, other important risk factors, namely mental illness, prior suicide attempts etc., were not considered in this study. These factors should not be neglected when we evaluate suicidal risk in clinical or non-clinical population using SER model or other AI techniques. Seventh, the emotional words were nominated by 10 professionals in suicide prevention, not based on words frequency of callers at high suicidal risk. It is not data-driven and, to some extent, is subjective approach. In future studies, the data-driven approach such as word frequency analysis could be taken into consideration.

## Conclusion

Emotional disturbance is an important risk factor for suicidal behavior and should be identified in a timely way for effective suicide prevention. In this study, the EWLSRA was developed and demonstrates adequate psychometric properties. This list could be useful for labeling suicide specific emotional characteristics of speech materials, which is a key step in the SER model, a novel technique for suicidal risk evaluation and identification.

## Data availability statement

The datasets presented in this article are not readily available because we inform that the tape-recordings included in this study are not publicly available due to the Information Security Regulation of the study site, however, other data, materials, and codes in this study are available from the corresponding author upon reasonable request. Requests to access the datasets should be directed to corresponding author.

## Ethics statement

The studies involving human participants were reviewed and approved by the Institutional Review Board of the Beijing Huilongguan Hospital. The ethics committee waived the requirement of written informed consent for participation.

## Author contributions

YT, GF, and JL: conceptualization. JS, SZ, YT, XD, and GF: methodology. JS, YT, and YY: formal analysis. JS, YT, XW, LZ, MW, YW, NL, and JW: investigation. JS, JW, and YT: writing – original draft preparation. JS, SZ, YT, XD, XW, GF, LZ, MW, YW, NL, JW, and JL: writing – review and editing. YT and JL: supervision. YT: project administration and funding acquisition. All authors agreed to the order of authors list and have read and approved the final manuscript for submission.
