# Supplementary material for: Establishment and psychometric characteristics of emotional words list for suicidal risk assessment in speech emotion recognition
Source: Front Psychiatry. 2022 Nov 11;13:1022036. doi: 10.3389/fpsyt.2022.1022036 (PMC9691664; doi:10.3389/fpsyt.2022.1022036)
Supplement: Supplementary file 1 [file Table_1.DOCX]

**Supplemental material**

Meaning of Suicide-related Emotional Words in the EWLSRA:

**Despair:** No hope, hopeless. Generally refers to the completely loss of faith in something. Hope was dashed, and it is impossible to achieve.

**Sadness:** A sad, heart-break emotion. Sadness is a negative basic emotion, usually referred to as an emotional reaction caused by separation, loss, and failure. It consists of emotional experiences of frustration, discouragement, depression and demoralization, etc.. The degree of sadness depends on the importance and value of what is lost; it also depends on the personal characteristics.

**Psychological Distress:** It is a widespread and disturbing feeling. It refers to psychological suffering, and mental torment pain. It is a state of psychological imbalance due to the elimination of hopes.

**Guilty:** It is a subjective feeling when a person does something which violates his or her conscience (the exact degree depends on the strengths of the conscience).

**Confusion:** A feeling of ambiguous or vague, or a state of mess. Someone do not know how to make a choice, no sense of direction.

**Helplessness:** While someone encounters difficulties, the person has a sense of powerlessness or could not cope with it.

**Resentment:** Includes anger and grudge. Anger not only refers to a tense and unpleasant emotion caused by frustration, but also feelings experienced while encountering some extremely disgusted events. Grudge referrs to strong dissatisfactions fill in mind, and emotional reactions to the dissatisfied others.

**Fear:** Scared, panicked, and terrified, while facing a dangerous situation, especially those could not perfectly solved.

**Numbness:** It refers to the numbness or even loss of sensation in mind. It is a metaphor for the lack of responsiveness or the lack of compassion to external events. A person with numbness experiences substantially different feeling from what happened in the outside real world.

**Anxiety:** An irritable emotion arising from excessive worry about the safety or fate of loved ones or oneself. It contains components such as anxiety, worry, nervousness, and uneasiness. It is associated with critical and unpredictable situations which are difficult to cope with.

**Grievance:** An emotion arises when someone is unduly blamed or treated.
